# Mobile Health App (DIGICOG-MS) for Self-Assessment of Cognitive Impairment in People With Multiple Sclerosis: Instrument Validation and Usability Study

**DOI:** 10.2196/56074

**Published:** 2024-06-20

**Authors:** Jessica Podda, Andrea Tacchino, Michela Ponzio, Federica Di Antonio, Alessia Susini, Ludovico Pedullà, Mario Alberto Battaglia, Giampaolo Brichetto

**Affiliations:** 1 Scientific Research Area Italian Multiple Sclerosis Foundation Genoa Italy; 2 Department of Physiopathology, Experimental Medicine and Public Health University of Siena Siena Italy

**Keywords:** cognitive assessment, cognitive impairment, digital health, mHealth app, multiple sclerosis, self-management, usability

## Abstract

**Background:**

Mobile health (mHealth) apps have proven useful for people with multiple sclerosis (MS). Thus, easy-to-use digital solutions are now strongly required to assess and monitor cognitive impairment, one of the most disturbing symptoms in MS that is experienced by almost 43% to 70% of people with MS. Therefore, we developed DIGICOG-MS (Digital assessment of Cognitive Impairment in Multiple Sclerosis), a smartphone- and tablet-based mHealth app to self-assess cognitive impairment in MS.

**Objective:**

This study aimed to test the validity and usability of the novel mHealth app with a sample of people with MS.

**Methods:**

DIGICOG-MS includes 4 digital tests assumed to evaluate the most affected cognitive domains in MS (visuospatial memory [VSM], verbal memory [VM], semantic fluency [SF], and information processing speed [IPS]) and inspired by traditional paper-based tests that assess the same cognitive functions (10/36 Spatial Recall Test, Rey Auditory Verbal Learning Test, Word List Generation, Symbol Digit Modalities Test). Participants were asked to complete both digital and traditional assessments in 2 separate sessions. Convergent validity was analyzed using the Pearson correlation coefficient to determine the strength of the associations between digital and traditional tests. To test the app’s reliability, the agreement between 2 repeated measurements was assessed using intraclass correlation coefficients (ICCs). Usability of DIGICOG-MS was evaluated using the System Usability Scale (SUS) and mHealth App Usability Questionnaire (MAUQ) administered at the conclusion of the digital session.

**Results:**

The final sample consisted of 92 people with MS (60 women) followed as outpatients at the Italian Multiple Sclerosis Society (AISM) Rehabilitation Service of Genoa (Italy). They had a mean age of 51.38 (SD 11.36) years, education duration of 13.07 (SD 2.74) years, disease duration of 12.91 (SD 9.51) years, and a disability level (Expanded Disability Status Scale) of 3.58 (SD 1.75). Relapsing-remitting MS was most common (68/92, 74%), followed by secondary progressive (15/92, 16%) and primary progressive (9/92, 10%) courses. Pearson correlation analyses indicated significantly strong correlations for VSM, VM, SF, and IPS (all *P*<.001), with r values ranging from 0.58 to 0.78 for all cognitive domains. Test-retest reliability of the mHealth app was excellent (ICCs>0.90) for VM and IPS and good for VSM and SF (ICCs>0.80). Moreover, the SUS score averaged 84.5 (SD 13.34), and the mean total MAUQ score was 104.02 (SD 17.69), suggesting that DIGICOG-MS was highly usable and well appreciated.

**Conclusions:**

The DIGICOG-MS tests were strongly correlated with traditional paper-based evaluations. Furthermore, people with MS positively evaluated DIGICOG-MS, finding it highly usable. Since cognitive impairment poses major limitations for people with MS, these findings open new paths to deploy digital cognitive tests for MS and further support the use of a novel mHealth app for cognitive self-assessment by people with MS in clinical practice.

## Introduction

Multiple sclerosis (MS) is a chronic, inflammatory, demyelinating, neurodegenerative disease of the central nervous system with an unpredictable clinical course and highly variable clinical manifestations [1]. It predominantly affects individuals in their early adult life and has a huge impact functionally, financially, and on quality of life [2]. The following 3 main clinical courses (phenotypes) of MS have been identified: relapsing-remitting MS (RRMS), secondary-progressive MS (SPMS), and primary-progressive MS (PPMS). These classifications are based on the presence or absence of activity, including relapses and progression, as well as on magnetic resonance imaging findings such as new lesions indicating inflammatory activity and atrophy suggesting ongoing neurodegeneration [2]. The most common MS symptoms are fatigue, pain, bladder and bowel issues, movement and coordination problems, visual problems, cognitive deficits, and emotional lability [2-5]. Although some symptoms are immediately obvious (eg, balance or gait disorders), others such as fatigue, mood disorders, and cognitive problems are often hidden, referred to as “invisible” symptoms.

Cognitive impairment, affecting about 43% to 70% of people with MS, is recognized as a prevalent and debilitating symptom. Cognitive impairment is documented in all MS courses, with more severe deficits in progressive forms, both SPMS and PPMS, compared with RRMS [6]. Multiple aspects of cognition can be affected in people with MS, including information processing speed (IPS), attention, executive functioning, working memory, and long-term memory [7-9]. Cognitive deficits negatively affect many life aspects for people with MS, including level of activity and participation in daily activities, including work and social life; increased risk of falls and car accidents; reduced interaction with health care providers; and decreased compliance with therapy and associated therapeutic benefits [10], and these difficulties could be grouped into homogenous and predominant cognitive phenotypes [11-13]. Although addressing cognitive impairment is recognized as a key priority in MS care that could lead to more timely and targeted treatment interventions, highly reliable and validated traditional paper-and-pencil tests such as the Brief International Cognitive Assessment for Multiple Sclerosis [14,15], Brief Repeatable Battery of Neuropsychological tests [16], and Minimal Assessment of Cognitive Function in MS [17] are still not widely and routinely used in clinical practice. Even in specialized centers, systematic cognitive assessments are somewhat time-consuming, require specialized examiners, and may not be well accepted by people with MS [18]. Moreover, as MS progresses, traditional paper-based tests may not detect subtle cognitive changes in a timely manner. Thus, these limitations may be overcome by alternative easy-to-use digital solutions.

Digital and remote technologies are being used to address challenges of great interest for the current and future research of MS, such as the improvement of safety, autonomy, and well-being during daily activities. Considering the high cost of providing health care to people with a neurological chronic disease and the increasing technological advancements in the rehabilitation field, the time is right to move from traditional, standard care center–based programs toward the implementation of innovative and alternative tools with high therapeutic benefits in routine practice [9].

Telemedicine has enabled convenient and effective follow-up visits, reduced unnecessary testing and referrals, maintained good perception of care, and, not least, reduced travel costs and caregiver burden [19]. The increasing use of smartphones and tablets has made mobile health (mHealth) apps a promising tool for empowering and engaging people in the self-management of their health [20]. In this scenario, mHealth apps can screen individuals who are at risk and offer self-help intervention or clinical referrals for various health conditions. They represent vehicles for enhanced real-time data capture to screen for, monitor, and treat cognitive impairment in MS and may fundamentally shift traditional paradigms of medicine [21-26]. However, although technology promises to offer people with MS alternative methods for tracking and self-managing symptoms, communicating with their health care providers, and improving their health-related quality of life, it is unclear if available mHealth tools are meeting their needs [27]. For instance, although Salimzadeh et al [20] found 104 MS-related apps in iTunes and Google Play, they noted that there was no corresponding evidence regarding the usability and utility of these solutions for people with MS. To be used in clinical practice, mHealth tools for cognition should be validated, and their usability and acceptance by end users should be tested.

Given the need to understand more in depth the potentiality of mHealth apps in the assessment of cognitive impairment in MS, we designed and developed DIGICOG-MS (Digital Assessment of Cognitive Impairment in Multiple Sclerosis), a smartphone and tablet-based app to self-assess cognitive impairment in people with MS. The aims of our study were to test the validity and usability of the novel mHealth app with a sample of people with MS. Furthermore, we evaluated whether demographic and clinical variables would explain any variability in usability and acceptability of DIGICOG-MS. We hypothesized that, (1) since consistent evidence indicates that scores obtained with traditional and electronic assessments do not differ [23,28], individuals’ performances on traditional paper-based and digital assessments would be highly comparable and (2) DIGICOG-MS would be well appreciated overall by people with MS.

## Methods

### Participants

The study sample consisted of people with MS enrolled by the Italian MS Foundation and receiving outpatient care at the Italian Multiple Sclerosis Society (AISM) Rehabilitation Service in Genoa (Italy). Potential eligible participants were identified by a trained neuropsychologist based on medical reports and were subsequently contacted by phone. Inclusion criteria were an age of 18 years and older; a confirmed MS diagnosis following the McDonald criteria [29]; any disease course (RRMS, SPMS, PPMS); the absence of relapsing in the last 3 months; an Expanded Disability Status Scale (EDSS) score [30] ≤7; and adequate visual, hearing, and motor capabilities to work on a tablet. Exclusion criteria were a Montreal Cognitive Assessment score 18, neurological and major psychiatric illness, past serious head trauma, and alcohol or drug abuse.

Upon enrollment and provision of written consent, each participant underwent 2 in-person evaluation sessions at the AISM Rehabilitation Service in Genoa, Italy. These sessions, each lasting approximately 30 minutes, were spaced apart by a convenient time interval of up to 10 days to minimize potential carryover learning effects. A trained neuropsychologist administered paper-based tests, while digital tests were self-administered by people with MS under supervision. The order of digital and paper-based assessments was counterbalanced.

Demographic information (age, gender, and education) and disease-related data (MS course, disease duration, and EDSS score) were collected for each participant. Furthermore, patient-reported outcomes (PROs) were assessed for self-reported mood (Hospital Anxiety and Depression Scale [HADS]) [31], upper-limb functionality (Arm Function in Multiple Sclerosis Questionnaire [AMSQ]) [32], and impact of MS (Multiple Sclerosis Impact Scale [MSIS-29]) [33]. Usability of the app was evaluated using the System Usability Scale (SUS) [34,35] and mHealth App Usability Questionnaire (MAUQ) [36], which were administered at the conclusion of the digital session. The 10-item SUS evaluates users’ personal perceptions about how to use a given system or device, ranging from “strongly disagree” (1 point) to “strongly agree” (5 points). Technology in general has an average score of 60 [37]. The SUS score ranges from 0 (lowest usability) to 100 (highest usability), with a value of 68 considered above average. The novel 18-item MAUQ is a validated and reliable questionnaire to assess mHealth apps. Each item’s score ranges from 1 to 7, which corresponds to the degree of agreement of the digital tool. The highest possible score is 126, and the lowest is 18, with an average total score ≥72 indicating that the app is usable [36]. This questionnaire has the following 3 subscales: ease of use, interface and satisfaction, and usefulness. Furthermore, the MAUQ is designed for different users (patients or health care providers) and different interaction modes (interactive or stand-alone). In stand-alone mHealth apps, the app users enter, collect, or store health information about themselves or other people. Stand-alone apps may generate reminders or show a summary or details about the collected health information, but these apps do not send the data to the user’s health care providers or to patients. Thus, for this study purpose, the stand-alone patient version of the scale was administered to people with MS. Data collection occurred between January 2023 and November 2023.

### DIGICOG-MS Tool

**Figure 1:**
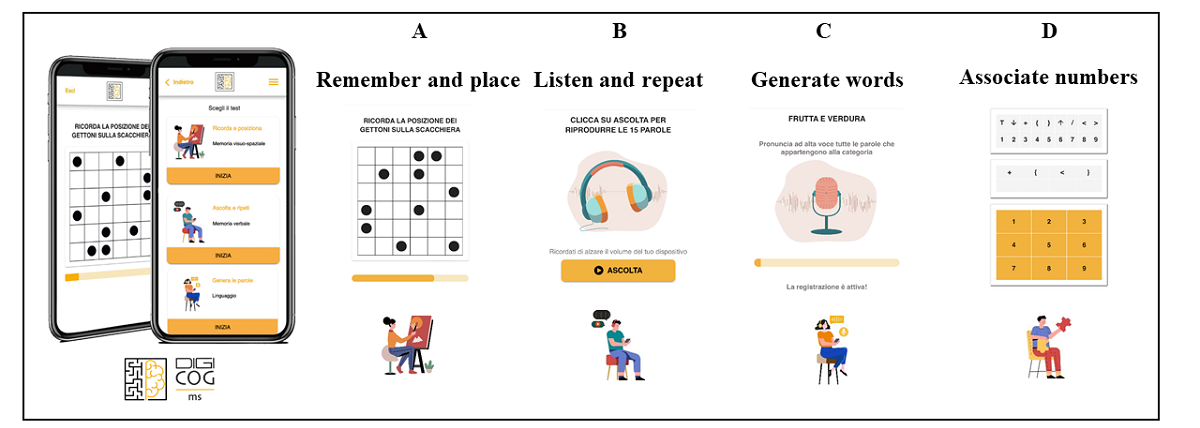
Visualization of the 4 digital tests in DIGICOG-MS mHealth app that assess (A) visuospatial memory, (B) verbal memory, (C) semantic fluency, and (D) information processing speed.

DIGICOG-MS (intellectual property of the Italian Multiple Sclerosis Foundation; SIAE Registration ID: D000018162, 27-12-2022) is an mHealth app developed to self-assess and monitor the presence of cognitive impairment (see Figure 1). The app, supported on Android and iOS, facilitates data collection, storage, and presentation, incorporating analysis algorithms and a clinician dashboard for user management and data extraction. The front end of the app was developed using the open-source framework Angular, a JavaScript-based TypeScript development language known for its efficiency and speed. A dedicated back-end service for DIGICOG-MS functionality was implemented using .Net Standard 6.

DIGICOG-MS integrated 4 digital tests (Remember and place, Listen and repeat, Generate words, Associate numbers) inspired by paper-based tests and assumed to measure the most affected cognitive domains in MS: visuospatial memory (VSM), verbal memory (VM), semantic fluency (SF), and IPS [38,39].

“Remember and place” assesses episodic VSM. A 36-square grid with 10 black checkers is displayed on the screen for 10 seconds. After the time elapses, the pattern disappears, and participants must reproduce it on a blank checkerboard. This replicates the 10/36 Spatial Recall Test (SPART) [39], in which a 6 × 6 checkerboard with 10 pieces arranged in a particular pattern is shown to the participant for 10 seconds. Both tests (digital and traditional) include 3 consecutive trials, and the score consists of the total number of correct responses for the 3 trials.

“Listen and repeat” was developed as an electronic version of the Rey Auditory Verbal Learning Test (RAVLT) [40], which evaluates VM. Participants listen to a prerecorded list of 15 common nouns and are asked to recall as many words as possible 5 times. Responses are recorded and scored by the neuropsychologist. In the traditional test, words are read aloud to the participant, who is asked to repeat as many words as possible in any order. All pronounced nouns in each of the 5 learning trials are transcribed by the neuropsychologist. For both versions of the test, the total score consists of the number of words recalled across the 5 trials.

“Generate words” is a digital adaptation of Word List Generation (WLG) [39] and measures verbal SF. Participants generate a list of words*,* typically constrained by a specific semantic category, in 90 seconds. Recordings of pronounced words are processed by the neuropsychologist for scoring. In the traditional test, all words generated within the given semantic category are transcribed by the neuropsychologist. The total score is based on the number of correct words produced.

“Associate numbers” represents a modified electronic version of the Symbol Digit Modalities Test (SDMT) [14,39], which measures attention and IPS. Participants are required to match single digits (1-9) to corresponding symbols as accurately and quickly as possible by clicking on a keyboard. A key pairing digits with symbols is available for the entire duration of the test, with a practice phase of 4 items provided to familiarize participants with the task. A 90-second timer is added to the system to avoid using a stopwatch and automatically terminates the procedure. In the traditional test, participants are presented with a page headed by a key of 9 symbols, and their task is to orally report the correct corresponding number. After completing the first 10 items with guidance, participants are timed to determine how many responses can be made in 90 seconds. In both versions of the test, the total score is based on the number of correct answers provided within 90 seconds. Additionally, in the digital test, the numbers of correct pairs at 30 seconds and 60 seconds are also provided.

Table 1 outlines the digital and traditional tests to assess VSM, VM, SF, and IPS. Although DIGICOG-MS tests were developed to closely mirror the original paper-based versions, minor adjustments were made during the digitalization process. Notably, substantial variations were implemented for the IPS digital test due to copyright restrictions associated with the SDMT (ie, different symbols and pairings have been settled). In this study, all people with MS used the app with Android OS. Although the current version of DIGICOG-MS is in the Italian language, the app was designed with scalability in mind, allowing for easy extension to other languages.

**Table 1:** Digital (delivered through DIGICOG-MS [Digital Assessment of Cognitive Impairment in Multiple Sclerosis]) and traditional tests administered to people with multiple sclerosis.

Cognitive domains	Digital	Traditional
Visuospatial memory	Remember and place	10/36 SPART^a^
Verbal memory	Listen and repeat	RAVLT^b^
Semantic fluency	Generate words	WLG^c^
Information processing speed	Associate numbers	SDMT^d^

^a^SPART: Spatial Recall Test.

^b^RAVLT: Rey Auditory Verbal Learning Test.

^c^WLG: Word List Generation.

^d^SDMT: Symbol Digit Modalities Test.

### Statistical Analysis

Categorical data are summarized with numbers and percentages, while numerical data are indicated by the mean and SD.

Convergent validity was computed using the Pearson correlation coefficient to determine the strength of the association between digital (ie, Remember and place, Listen and repeat, Generate words, Associate numbers) and traditional (ie, 10/36 SPART, RAVLT, WLG, and SDMT) tests. Pearson correlation was considered as strong (r≥0.5), moderate (0.5>r>0.3), and weak (0.3>r>0.1) [41]. Known as a common tool to compare 2 methods of measurement to determine the overall degree of agreement, Bland-Altman scatterplots were created to estimate the disagreement between traditional and digital tests as a function of the mean of the 2 measurements for each cognitive domain. On the y axis, the difference between every pair of measurements for each cognitive domain is plotted, and the x axis shows the mean of these measures. Horizontal reference lines on the plot represent the average difference between the measurements (dotted line), which is expected to be 0 for a good level of agreement, and limits of agreement (sharp lines), which are fitted at ±1.96 SD. When there was only a single score without a pair (ie, a digital or traditional assessment was missing), the participant was excluded from the final analysis.

The test-retest reliability of DIGICOG-MS was assessed using intraclass correlation coefficients (ICCs) in a cohort of people with MS 2 weeks apart from the first digital evaluation. Using power analysis calculations to test reliability between 2 different observations as described by Bujang and Baharum [42], a minimum of 22 individuals was needed to have an acceptable ICC value ≥0.5 (α=.05, power=80%, n=2). A 2-week window was chosen to reduce variability that is independent of general disease status and might be attributed to good or bad days or to differences between weekdays and weekends [23]. A 2-way mixed effects model on absolute agreement for single measurements was used. Reliability was considered as excellent (ICC≥0.9), good (ICC≥0.75), acceptable (0.75>ICC≥0.5), or poor (ICC<0.5) [43].

To investigate potential factors influencing the usability as measured by SUS and MAUQ, univariate linear regression analysis was conducted using a selection of predictors from demographic (ie, age and educational level) and clinical (ie, EDSS, MS course, MS duration, HADS-A [anxiety subscale], HADS-D [depression subscale], MSIS-29, and AMSQ) variables. Min-max rescaling was previously applied to PRO items. The median values were used to fill in the missing values. All confidence intervals were 2-sided and used 95% confidence levels. *P* values <.05 were considered statistically significant. Statistical analysis was performed using STATA (version 17) and MATLAB (version 9.14.0 [R2023a] Update 4).

### Ethical Considerations

The study was approved by the Regional Ethics Committee of Azienda Ospedaliera “San Martino” of Genoa, Italy (number 240/2022 - DB id 12354), and conducted according to the Declaration of Helsinki [44]. Before entering the study, participants had to read, complete, and sign an informed consent form. Data collected were stored in an anonymized format to properly protect the privacy and confidentiality of participants, ensuring that no individual can be identified from the data provided. Participants were informed that data collection could be used only for research purposes.

## Results

### Participants

At the beginning, 105 people with MS met the eligibility criteria and were contacted by the neuropsychologist. The first evaluation was postponed by 7 people with MS due to multiple reasons (eg, health problems, travel constraints) several times until the neuropsychologists decided to withdraw them from the study. We enrolled 98 people with MS and excluded 6 participants from the analyses due to a missing score on 1 assessment (ie, digital or traditional modality). The final sample consisted of 92 people with MS (60 women). A summary of their demographic and clinical characteristics is reported in Table 2.

**Table 2:** Demographic and clinical sample characteristics (N=92).

Characteristic			Value
Age (years), mean (SD)			51.38 (11.36)
Age (years), range			(20-69)
**Gender, n (%)**			
	Female	60 (65)	
	Male	32 (35)	
Education (years), mean (SD)			13.07 (2.74)
Education (years), range			8-22
Disease duration (years), mean (SD)			12.91 (9.51)
Disease duration (years), range			1-32
**Disease course, n (%)**			
	RRMS^a^	68 (74)	
	SPMS^b^	15 (16)	
	PPMS^c^	9 (10)	
EDSS^d^, mean (SD)			3.58 (1.75)
EDSS, range			1-7.5
**HADS^e^, mean (SD)**			
	HADS-D^f^	5.87 (4.17)	
	HADS-A^g^	7.6 (4.9)	
**HADS, range**			
	HADS-D	0-19	
	HADS-A	0-19	
AMSQ^h^, mean (SD)			54.14 (28.10)
AMSQ, range			31-136
MSIS-29^i^, mean (SD)			65.9 (27.6)
MSIS-29, range			2-136

^a^RRMS: relapsing-remitting multiple sclerosis.

^b^SPMS: secondary progressive multiple sclerosis.

^c^PPMS: primary progressive multiple sclerosis.

^d^EDSS: Expanded Disability Status Scale.

^e^HADS: Hospital Anxiety and Depression Scale.

^f^HADS-D: Hospital Anxiety and Depression Scale-depression subscale.

^g^HADS-A: Hospital Anxiety and Depression Scale-anxiety subscale.

^h^AMSQ: Arm Function in Multiple Sclerosis Questionnaire.

^i^MSIS-29: Multiple Sclerosis Impact Scale 29.

### Correlation Between Digital and Traditional Assessment Tests

**Figure 2:**
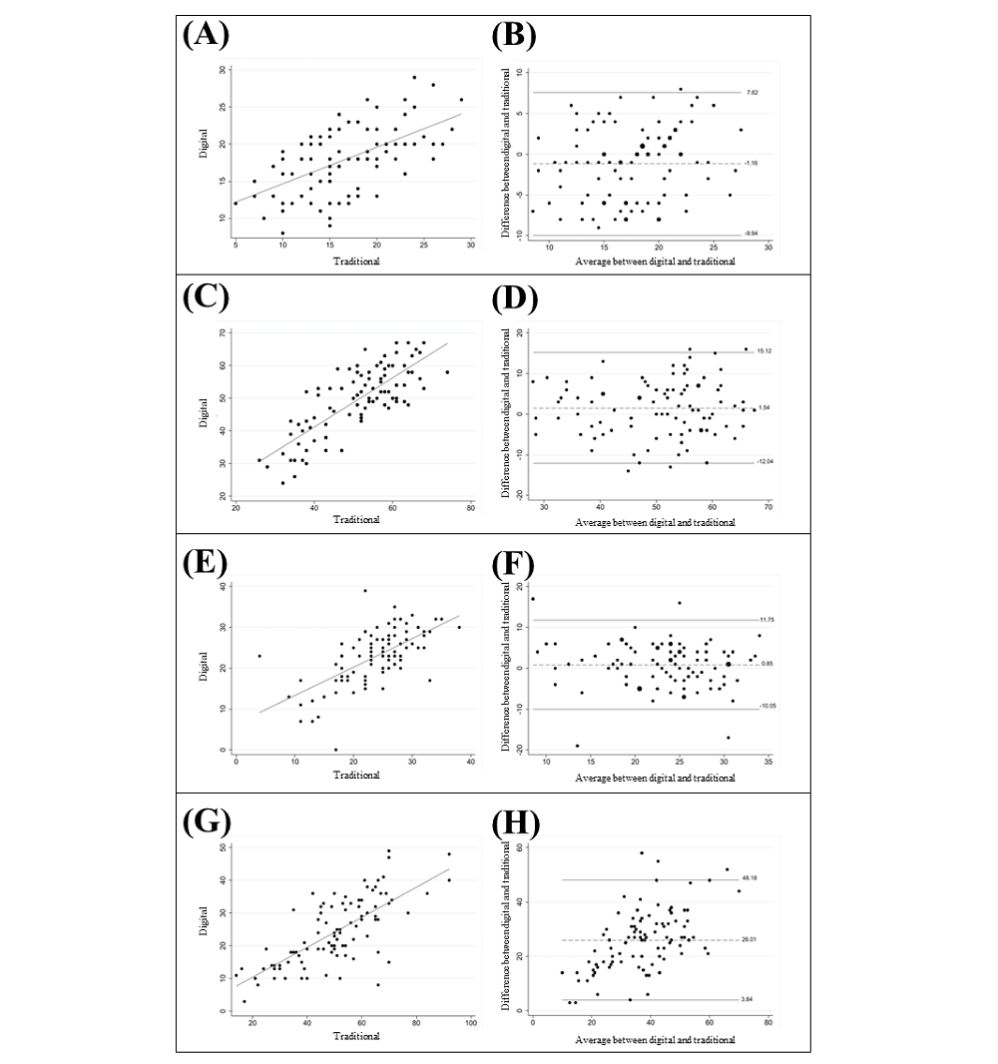
Scatterplots showing the correlations and Bland-Altman plots (dashed line: mean difference and should align with y=0 for optimal agreement; solid lines: 95% limits of agreement, within with all points should fall) comparing digital and traditional assessment tests for the following cognitive domains frequently altered in multiple sclerosis: (A) and (B) visuospatial memory (digital: Remember and place; traditional: 10/36 Spatial Recall Test), (C) and (D) verbal memory (digital: Listen and repeat; traditional: Rey Auditory Verbal Learning Test), (E) and (F) semantic fluency (digital: Generate words; traditional: Word List Generation), (G) and (H) information processing speed (digital: Associate numbers; traditional: Symbol Digit Modalities Test).

The Pearson correlation analysis indicated significantly strong correlations for VSM, VM, SF, and IPS (all *P*<.001), with associations ranging from 0.58 to 0.78 (see Table 3 and Figure 2). The Bland–Altman plot for each cognitive domain showed a larger systematic bias for IPS, with a mean difference of –26.01 (limits of agreement: 3.84 to –48.18). For VM and SF, the mean difference was close to 0 (1.54 and 0.85, respectively), indicating that digital scores were slightly larger than traditional scores, while for the VSM, a negative mean difference was observed (–1.16; Figure 2).

**Table 3:** Correlation coefficients (Pearson *r* and 2-tailed *P* value) between digital and traditional tests.

Variable		VSM^a^ traditional	VM^b^ traditional	SF^c^ traditional	IPS^d^ traditional
**VSM digital**					
	r	0.580	0.454	0.248	0.526
	*P* value	<.001	<.001	.02	<.001
**VM digital**					
	*r*	0.439	0.785	0.405	0.649
	*P* value	<.001	<.001	<.001	<.001
**SF digital**					
	*r*	0.465	0.392	0.636	0.625
	*P* value	<.001	<.001	<.001	<.001
**IPS digital**					
	*r*	0.518	0.519	0.539	0.720
	*P* value	<.001	<.001	<.001	<.001

^a^VSM: visuospatial memory.

^b^VM: verbal memory.

^c^SF: semantic fluency.

^d^IPS: information processing speed.

### Test-Retest Reliability

Test-retest reliability values of the mHealth app were obtained from a randomly selected subset of people with MS (n=27). Results showed excellent reliability for VM and IPS (ICCs≥0.95) and good reliability for VSM and SF (ICCs≥0.83). Scores from digital tests in the DIGICOG-MS increased during the second test after 2 weeks, likely due to practice effects. Results of the test-retest reliability are summarized in Table 4.

**Table 4:** The test-retest reliability of DIGICOG-MS (Digital Assessment of Cognitive Impairment in Multiple Sclerosis) tests in 4 cognitive domains (n=27).

Variable	Time 1, mean (SD)	Time 2, mean (SD)	Mean difference (SD)	ICC^a^ (95% CI)
VSM^b^	17.6 (4.8)	18.7 (4.7)	1.11 (2.50)	0.84 (0.67-0.93)
VM^c^	50.4 (11.2)	51.9 (10.9)	1.52 (2.76)	0.96 (0.89-0.98)
SF^d^	22.8 (7.6)	23.8 (6.9)	1.04 (4.12)	0.83 (0.67-0.92)
IPS^e^	23.9 (11.4)	25.3 (10.6)	1.44 (3.08)	0.95 (0.89-0.99)

^a^ICC: intraclass correlation coefficient.

^b^VSM: visuospatial memory.

^c^VM: verbal memory.

^d^SF: semantic fluency.

^e^IPS: information processing speed.

### DIGICOG-MS Usability Testing

The average SUS score was 84.5 (SD 13.34), indicating “best imaginable” usability (range 84.1-100) [45]. The average total MAUQ score was 104.02 (SD 17.69), well above the cut-off of 72, suggesting that DIGICOG-MS was significantly usable and well appreciated by people with MS [36]. Average subscale scores were as follows: ease of use, 30.57 (SD 5.18); interface and satisfaction, 42.96 (SD 7.93); usefulness, 30.49 (SD 7.14). Depressive symptoms, as indicated by the HADS-D, were negatively correlated with both MAUQ (r=–0.31, *P*<.001) and SUS (r=–0.17, *P*=.02) and show the major effects on usability outcome measures compared with all other covariates; anxiety, as indicated by the HADS-A, was only associated with MAUQ (r=–0.16, *P*=.04), with a minor but confirmative effect. See Table 5 for a summary of total usability scores from the SUS and MAUQ.

**Table 5:** Associations between usability as indicated by total System Usability Scale (SUS) and mHealth App Usability Questionnaire (MAUQ) scores and demographic and clinical variables.

Variable	Total MAUQ score			Total SUS score	
	Coefficient (SE)	*P* value	Coefficient (SE)		*P* value
Age	–0.0015 (0.0014)	.28	–0.0021 (0.0012)		.09
Years of education	0.005 (0.006)	.41	0.010 (0.005)		.05
MS^a^ course (RR^b^)	0.013 (0.04)	.76	0.033 (0.038)		.39
Gender (male)	–0.006 (0.032)	.85	0.039 (0.029)		.19
Disease duration	–0.0014 (0.0016)	.37	–0.0022 (0.0015)		.14
EDSS^c^	0.007 (0.009)	.45	–0.006 (0.008)		.50
MSIS-29^d^	–0.10 (0.07)	.19	–0.11 (0.06)		.08
AMSQ^e^	–0.145 (0.096)	.13	–0.15 (0.08)		.05
HADS-A^f^	–0.16 (0.07)	.04	–0.11 (0.06)		.08
HADS-D^g^	–0.31 (0.08)	<.001	–0.17 (0.07)		.02

^a^MS: multiple sclerosis.

^b^RR: relapsing-remitting.

^c^EDSS: Expanded Disability Status Scale.

^d^MSIS-29: Multiple Sclerosis Impact Scale.

^e^AMSQ: Arm Function in Multiple Sclerosis Questionnaire.

^f^HADS-A: Hospital Anxiety and Depression Scale-anxiety subscale.

^g^HADS-D: Hospital Anxiety and Depression Scale-depression subscale.

A higher level of education was positively correlated with the MAUQ ease of use subscale (r=0.012, *P*=.03), while EDSS was correlated with the usefulness subscale (r=0.025, *P*=.03). Both anxiety and depression appeared to have a significant impact on mHealth app usability. HADS-D score was inversely proportional to all MAUQ subscales (ease of use: r=–0.20, *P*=.03; interface and satisfaction: r=–0.33, *P*=.001; usefulness: r=–0.37, *P*<.001); HADS-A score was inversely proportional to the interface and satisfaction subscale only (r=–0.21, *P*=.02). Interestingly, upper limb functionality on the AMSQ had an important negative relationship (r=–0.25) with the individual evaluation of the interface and satisfaction subscale (*P*=.02). Except for HADS-D score, neither clinical nor demographic variables were correlated with the SUS score (see Table 6 for a detailed overview of the MAUQ subscale results).

**Table 6:** Correlations between the mHealth App Usability Questionnaire (MAUQ) subscales and demographic and clinical variables.

Variable	Ease of use MAUQ subscale			Interface and satisfaction MAUQ subscale			Usefulness MAUQ subscale	
	Coefficient (SE)	*P* value	Coefficient (SE)		*P* value	Coefficient (SE)		*P* value
Age	–0.0025 (0.0013)	.07	–0.0020 (0.0015)		.21	–0.0001 (0.0018)		.97
Years of education	0.0123 (0.005)	.03	0.003 (0.006)		.62	0 (0.008)		.99
MS^a^ course (RR^b^)	0.014 (0.04)	.73	0.01 (0.05)		.86	0.02 (0.06)		.78
Gender (male)	0.001 (0.032)	.97	–0.013 (0.037)		.73	–0.01 (0.04)		.91
Disease duration	–0.0025 (0.0016)	.13	–0.0016 (0.0018)		.40	–0.0005 (0.0022)		.83
EDSS^c^	–0.006 (0.009)	.51	0 (0.010)		.98	0.025 (0.012)		.03
MSIS-29^d^	–0.08 (0.08)	.32	–0.14 (0.09)		.11	–0.07 (0.09)		.45
AMSQ^e^	–0.12 (0.10)	.24	–0.25 (0.11)		.02	–0.04 (0.12)		.72
HADS-A^f^	–0.11 (0.08)	.16	–0.21 (0.09)		.016	–0.13 (0.09)		.18
HADS-D^g^	–0.20 (0.09)	.03	–0.33 (0.10)		.001	–0.37 (0.10)		<.001

^a^MS: multiple sclerosis.

^b^RR: relapsing-remitting.

^c^EDSS: Expanded Disability Status Scale.

^d^MSIS-29: Multiple Sclerosis Impact Scale.

^e^AMSQ: Arm Function in Multiple Sclerosis Questionnaire.

^f^HADS-A: Hospital Anxiety and Depression Scale-anxiety subscale.

^g^HADS-D: Hospital Anxiety and Depression Scale-depression subscale.

## Discussion

### Principal Findings

This study aimed to determine whether DIGICOG-MS, an mHealth app for self-cognitive assessment including digital tests for VSM, VM, SF, and IPS, could effectively evaluate cognitive impairment in adults with MS. Overall, the findings revealed strong correlations between digital and traditional paper-based tests across all cognitive domains, with correlation coefficients (r) ranging from 0.58 to 0.78. Test-retest reliability was excellent for VM and IPS (ICCs≥0.95) and good for VSM and SF (ICCs≥0.83). Additionally, people with MS positively evaluated the DIGICOG-MS app, finding it highly usable.

### Comparison With Prior Work

For VSM, no previous studies on digital SPART assessment in MS are currently available. However, van Dongen et al [22] tested an electronic version of the SPART with a group of 235 healthy participants, reporting good consistency for digital and traditional tests. Although significant (r=0.58), the correlation between SPART and the “Remember and place” task was lower than with the other cognitive domains investigated. One speculation could be the intrinsic heterogeneity in the task procedure: In the paper-based version, participants had to pick and move black coins in the correct position on the checkerboard, while in the digital test, individuals had to tap on the empty grid. Although a swipe command is more similar to the gesture of pick and place [22], we decided to ask participants to click on the screen to avoid any difficulty experienced by participants in placing pressure on the screen device.

Regarding the VM domain, our results are in line with those of a previous study that developed an electronic version of RAVLT with people with MS [21]. However, in the study by Beier et al [21], the word list was available on the screen, and as the participants verbally recalled words, a single tap on each word recorded a correct response. The highly significant correlation (r=0.78) observed in our study reflects that the digital version resembles the traditional version in terms of stimuli administration and scoring procedures.

A similar pattern was observed for the SF task. To our knowledge, “Generate words” is the first digital test to evaluate fluency for semantic stimuli in MS. A strong correlation with the paper-based versions of the WLG (r=0.64) was observed, reflecting excellent similarity in the administration procedure between the 2 modalities.

Notably, although the correlation between traditional and digital tests for IPS was strong (r=0.72) and comparable with previously reported associations between smartphone-based and paper-based versions of the SDMT [23,46], individual performances on the “Associate numbers” test tended to be lower than those on the oral SDMT. In a recent study by Costabile et al [25] that tested the potentiality of the tablet version of the Brief International Cognitive Assessment for Multiple Sclerosis, authors found a very small mean difference between the 2 administration modalities (mean –2.72). However, they administered an electronic SDMT as if it was a paper-based test and used the iPad as a back end for neuropsychologists to record the answers given orally by participants. Our decision to implement a written version for the digital IPS test was to allow people with MS to complete the task in a fully unsupervised and autonomous way. Montalban et al [23] reported several possible sources of performance discrepancy. First, in the digital test, a longer time was required to tap the correct response on a keyboard compared with saying the correct response aloud. Second, the digital IPS test displayed 4 symbols at a time, while, in contrast, the SDMT provides participants the entire symbol key printed on a sheet of paper, leading to massive involvement of eye tracking and visual working memory. In addition, since the SDMT is recognized as the recommended screening tool for cognitive impairment in MS [47], most people with MS may have familiarity with this test and thus have memorized the symbol-digit code of the paper-based test.

Although the potential benefits of digital tools in terms of assessment and prediction of cognitive changes over time, and thus to plan a tailored rehabilitation intervention, have been confirmed, the usability of the proliferating mHealth apps is sometimes unknown and under-investigated [48]. The majority of studies investigating the potential of novel digital tools for assessment purposes missed or did not report a detailed usability examination [21,22,25]. A meaningful mHealth app for people with MS needs to be usable, useful, and satisfying; thus, understanding its usability provides insight into the quality and overall satisfaction of the user’s experience. In our study, people with MS positively evaluated DIGICOG-MS, finding it highly usable and motivating.

Overall, an inverse correlation between depressive symptoms and usability was found. Compared with other covariates, these symptoms exhibit the greatest effect strength, closely followed by anxiety. This is in line with the findings by Ly et al [49], who noted the key impact of major depressive illness on a participant’s ability to interact with mHealth apps in general. Since the user experience of a digital tool is crucial, especially as it might be relevant in avoiding dropout, the biased impression of a person with depressive symptoms and resulting needs of those users should be considered when designing and evaluating digital (mental) health platforms [50]. The literature supports this concern, with Torous et al [51] advising that many apps are not designed with the end user primarily in mind and that apps are unhelpful in emergencies. They reported a study by Sarkar et al [52] in which more than 50% of participants with major depressive disorder had some degree of difficulty both entering and accessing their mood data across 4 common and popular mood tracking apps.

As expected, upper limb functionality correlated with the interface and satisfaction subscale, confirming that upper limb limitations can also prevent people, especially older adults, from using mHealth apps [53]. Indeed, the degree of influence exerted by upper limb functionality on the appreciation of the benefits provided by the digital tool is substantial and comparable to that of the mood domain. The correlation between the ease of use subscale and educational level is in line with that found by Marrie et al [54], who noted that a higher educational level is associated with a greater likelihood of mHealth app use. Since the app was rated as having, overall, the “best imaginable” levels of usability, our findings suggest that such a positive experience could increase the willingness to use DIGICOG-MS, leading to greater adherence to future interventions proposed by clinicians. It is important to acknowledge that involving end users in different phases of app development (ie, conception, design, and testing), working closely with them to learn about their needs, and formulating how an app may even be of use represent a useful approach to increase usability and engagement.

### Limitations and Strengths

We acknowledge some limitations of this study and suggest avenues for future research.

First, although the digital tests in the DIGICOG-MS were developed to be as similar as possible to the original paper-based versions, some changes in digital test development could affect participants’ performance. In this study, a fully automated, modified version of the SDMT was proposed that required people with MS to tap the correct answer using a number keyboard. However, we believe that an oral version of such a digital test will minimize the influence of hand disability on task performance [55,56] and thus accommodate more characteristics of people with MS [57]. Future developments of DIGICOG-MS will include integration with speech recognition software, allowing for fully automated use. This implementation will further provide an automatic score for those tests that require speech registration (ie, VSM and VM tests). To ensure proper clinical implementation of DIGICOG-MS as a screening tool, a sample of healthy participants should be enrolled in a further study to obtain norms for each digital test, enabling interpretation in a clinical setting (eg, indicate normal functioning or the presence of mild, moderate, or severe impairment).

Second, the digital assessment was conducted on site under the guidance of a trained neuropsychologist. Although this approach may have some limitations in terms of generalizability to real-life scenarios, it was a deliberate choice. Our sample lacked prior experience with digital tools for remote cognitive assessment. Thus, we opted to conduct validation and usability tests in a controlled clinical setting. This allowed participants to familiarize themselves with the novel technology and provided us with valuable feedback to enhance the app’s quality.

Moving forward, our future research endeavors will involve deploying DIGICOG-MS in a more realistic environment, such as the homes of people with MS. This approach will enable us to explore the tool’s potentialities in a setting that more closely mirrors everyday life, thus addressing concerns regarding generalizability.

Third, as a single-center study, the heterogeneity of the personal and clinical characteristics of our sample, ranging from younger, active, and autonomous individuals to older, sedentary, and dependent people, leads to a wide spectrum of symptoms and different needs [58]. Participants’ characteristics may limit the interpretation of our results in terms of both validity and usability. The study sample may be considered representative of clinic-attending people with MS followed as outpatients in rehabilitation centers (ie, middle-age or older adults and with a longer disease duration) [59]. Thus, results may not generalize to other populations of individuals with MS (eg, young and neodiagnosed people).

Fourth, it is known that previous experience with technologies increases acceptance for technological approaches, and some older adults could have less experience or interest in using their smartphone regularly than younger individuals with MS. This aspect should be taken into account when designing and developing such a digital tool for a wide variety of needs and characteristics of people with MS [54].

As a digital and remote communication technology app, DIGICOG-MS was developed for clinical monitoring and self-evaluation, as an alternative or complementary to traditional in-clinic assessments that could be implemented rapidly and routinely to address cognitive impairment in MS. To our knowledge, this is the first novel mHealth app developed for tablets and smartphones in the Italian language and validated in a sample of people with MS (available for download from Google Play store or Apple Store soon). Ultimately, although this version will be susceptible to changes, it could be carried out at the homes of people with MS, helping those people who are unable to access clinics easily for various reasons such as mobility restrictions, travel costs, consultation and treatment time constraints, and a lack of locally available expert MS services [60]. This will effectively reduce the distance between patients and their care professionals.

A self-evaluated cognitive tool, like DIGICOG-MS, brings potential advantages. First, digital administration procedures are more standardized than in the paper-based versions, reducing inter-rater differences and assessor-dependent errors in stimuli presentation (eg, prerecorded audio with an appropriate pace and tone could avoid prosody inflections that would suggest the position of the word within the list in the VM test), thus improving accuracy. Second, an app could reduce high stress levels and demands for clinicians resulting from repeating administration procedures over time. This aligns with results from previous studies that highlighted the potentialities of a digital tool for cognitive assessment in MS [26,61,62]. As indicated by Tacchino et al [63], digital cognitive assessments, which are expected to be conducted more frequently, at home or in an unsupervised setting could also support integration with electronic PROs of other dimensions significant for MS (eg, mood). Brichetto et al [64] found that PROs and clinician-assessed outcomes could be used to build accurate models of MS disease course prediction (ie, transition between a relapsing-remitting form to a secondary progressive form). In addition, PROs and clinician-assessed outcomes, alone or integrated with other indexes such as magnetic resonance imaging outcomes and biomarkers, could help the decision-making process of clinicians in their daily practice. The availability of longitudinal, multidomain, big data could allow the application of revolutionary technology, such as a digital twin, to MS cognitive phenotyping [65-67], where analysis of big data through artificial intelligence enables visualization of a virtual copy (twin) of the patient at different stages of the disease and supports further therapeutic decisions [68]. In conclusion, the possibility to better define the level of clinical complexity of each individual with MS and have sufficient and adequate predictive criteria for MS evolution through a novel digital solution like DIGICOG-MS could be pivotal for the construction of a more fruitful therapeutic pact between patient and clinician based on better perspective knowledge, increased disease consciousness, and enhanced engagement in rehabilitative treatments.

### Conclusion

Since cognitive impairment poses major limitations to people with MS, the current results open new paths to deploying digital cognitive tests for MS and further support the use of DIGICOG-MS for cognitive self-assessment in people with MS into clinical practice. Follow-up measurements will be easier to implement and could lead to timely identification of cognitive decline in people with MS and subsequently allow for adequate counseling. Deeper knowledge on cognitive phenotypes to detect cognitive impairment early, more accessible and tailored interventions, and better understanding of multitasking deficits in everyday life activities should be considered the main goals by current and future research in MS. Thus, an mHealth app such as DIGICOG-MS could be one of the available technological solutions necessary to address these goals.

## Abbreviations

AISM: Italian Multiple Sclerosis Society
AMSQ: Arm Function in Multiple Sclerosis Questionnaire
DIGICOG-MS: Digital Assessment of Cognitive Impairment in Multiple Sclerosis
EDSS: Expanded Disability Status Scale
HADS-A: Hospital Anxiety and Depression Scale-anxiety subscale
HADS-D: Hospital Anxiety and Depression Scale-depression subscale
ICC: intraclass correlation coefficient
IPS: information processing speed
MAUQ: mHealth App Usability Questionnaire
mHealth: mobile health
MS: multiple sclerosis
MSIS-29: Multiple Sclerosis Impact Scale
PPMS: primary progressive multiple sclerosis
PRO: patient-reported outcome
RAVLT: Rey Auditory Verbal Learning Test
RRMS: relapsing-remitting multiple sclerosis
SDMT: Symbol Digit Modalities Test
SF: Semantic Fluency
SPART: 10/36 Spatial Recall Test
SPMS: secondary progressive multiple sclerosis
SUS: System Usability Scale
VM: verbal memory
VSM: visuospatial memory
WLG: Word List Generation

## Data Availability

All data produced in this study are available upon reasonable request to the corresponding author.
